# Effects of ultrasound-assisted freezing and high-voltage electric field thawing on quality of precooked duck meat

**DOI:** 10.3389/fnut.2026.1791662

**Published:** 2026-04-24

**Authors:** Yingjuan Wei, Fengming Chen, Tingting Xie, Zhihong Sun, Meijuan Yu, Hui Yang, Kelang Kang

**Affiliations:** 1 Hunan Institute of Agricultural Product Processing and Quality Safety, Hunan Academy of Agricultural Sciences, Changsha, China; 2 Hunan Provincial Key Laboratory of the Traditional Chinese Medicine Agricultural Biogenomics, Changsha Medical University, Changsha, Hunan, China; 3 Hunan Arui Food, Juewei Food Co., Ltd, Changsha, China; 4 Yuelushan Laboratory, Changsha, China

**Keywords:** high-voltage electrostatic field-assisted thawing, microstructure, precooked meat, ultrasonic freezing, water migration

## Abstract

Flavor and textural changes are major quality challenges for prepared dishes, especially for precooked meat. However, studies mainly focused on raw meat and have not comprehensively evaluated integrated freezing and thawing methods. In this study, the effects of quick freezing with ultrasonic (QFU, 4.41 cm/h) combined with high-voltage electrostatic field-assisted thawing (HVEF) on the quality of precooked duck meat were investigated, in comparison to traditional freezing, including −18 °C freezing (CON, 1.0 cm/h), −40 °C quick freezing (QF, 2.07 cm/h), liquid nitrogen freezing (LNF, 8.16 cm/h) and thawing methods, including high-capacity refrigerator (HR), cool water flowing (CWF), and room temperature thawing (RT). The results demonstrate that with a higher freezing rate, QFU reduced the centrifugal loss of meat by 17.2%, improved textural attributes, and depressed the lipid and protein oxidation. Scanning electron microscopy showed that QFU and LNF produced orderly ice-crystal structures, with 70.5% of LNF ice crystals smaller than 12 μm^2^. In addition, faster freezing rates were associated with a lower proportion of T23 water. The HVEF thawing rendered the textural properties of precooked meat closer to those achieved by CWF. The HR thawing resulted in a lower free water content in precooked meat, despite the longest thawing time. These results provide a reference for maintaining quality of precooked meat through the industrial supply chain.

## Introduction

Precooked dishes incorporate semi-prepared and ready-to-eat products sourced from a variety of agricultural origins, whose market has experienced significant expansion in China in recent years (1). Precooked meat is a kind of precooked dish, consisting of intricate processing and multiple attributes. It offers benefits such as convenience through shorter cooking times, versatility with numerous meat choices, and quality using premium meat ingredients (2). Precooked meat products are characterized by high fat and protein content, making them highly susceptible to oxidative deterioration during processing and storage. Beyond flavor and texture, oxidative changes also degrade nutritional value by destroying essential fatty acids and amino acids (3). Furthermore, precooked meat is vulnerable to pathogens such as *Listeria monocytogenes* (4), making cold chain integrity essential for food safety. The texture and flavor are primary drivers of consumer acceptability, quality deterioration not only reduces nutritional and sensory value but also leads to significant economic losses through product waste and reduced market competitiveness. Therefore, understanding and optimizing freezing and thawing processes is critical for maintaining the quality, safety, and commercial viability of precooked meat products throughout the industrial supply chain.

Both freezing treatments and post cold chain thawing procedures significantly influence the quality of precooked meat. Emerging freezing technologies to enhance meat quality include ultra-low temperature freezing, liquid nitrogen freezing, immersion freezing, ultrasonic assisted immersion freezing, and HVEF freezing (5). These advanced freezing methods effectively reduce the phase transition duration and enhance the degree of supercooling, leading to the formation of smaller, more regularly shaped, and uniformly distributed ice crystals (6), which results in higher quality for meat. Ultrasound-assisted freezing is an innovative and environmentally friendly technology. For instance, Wu et al. (7) reported that under the ultrasonic assisted process with power density of 80 W/L, the pork samples had protected microstructure and water distribution, and quality deterioration were suppressed. The pork longissimus muscle treated with 180 W ultrasonic freezing were also reported to exhibit smaller and more uniformly distributed ice crystals, reduced thawing and cooking losses, decreased lipid oxidation, and improved redness and shear force (8). These benefits are primarily attributed to the microstreaming effects generated by the oscillation of ultrasound-induced cavitation bubbles, which enhance convective heat transfer, expedite the freezing process, and concurrently fragment existing ice crystals into smaller particles (9). An appropriate thawing method is also important in meat industry. Thawing methods include traditional approaches such as thawing at room temperature and cold-water thawing, as well as accelerated techniques based on physical and energy fields, including microwave thawing, radiofrequency thawing, electrostatic field thawing, and magnetic field-assisted thawing (5). These emerging technologies help preserve the quality of meat products. Striking a balance between thawing efficiency and post-thaw quality is interesting, and HVEF thawing has the potential (10). For instance, Hu et al. (11) applied a high-voltage electric field to thaw beef and pork, which reduced tissue damage during freezing and thawing cycle and achieved a higher proportions of *α*-helix and *β*-sheet structures in the thawed meat by preserving protein structure and properties. Amiri et al. (12) investigated the effects of a 10 kV HVEF on the thawing of beef and bovine myofibrillar proteins, finding that an increase in the number of needle electrodes decreased thiobarbituric acid values, thawing loss, drip loss and cooking loss. The mechanism by which a HVEF accelerates ice melting is attributed to the micro-level energy generated by the field, which enhances the disruption of hydrogen bonds within the ice lattice, promoting the formation of small ice crystals that subsequently transition into liquid water.

Most previous studies focused on the effects of individual freezing or thawing methods on the quality attributes of frozen meat. In contrast, only a limited number of studies have explored synergistic strategies of freezing and thawing approaches. Integrating different freezing and thawing technologies can address and ameliorate the limitations of single techniques (13). Furthermore, previous research on freezing and thawing typically focused on raw meats, which reflects scenarios pertinent to the long-distance transportation between slaughterhouses and processing facilities. During cooking, the heat-induced denaturation of myofibrillar proteins and the loss of loosely bound water have already altered the structural and water-holding characteristics of the meat. Consequently, the freezing and thawing responses of precooked meat differ from those of raw meat. However, there are relatively few studies in precooked meat subjected to rapid freezing and thawing processes.

This study investigated the effects of ultrasound-accelerated freezing in conjunction with high-voltage electrostatic field-assisted thawing on the quality of precooked duck meat, in comparison to traditional freezing and thawing methods. Furthermore, the mechanisms for changes in freshness and moisture loss of precooked meat were elucidated, which provides scientific insights and data for the industrial production of precooked meat dishes.

## Materials and methods

### Preparation of precooked duck meat samples

The cooking method and process have been standardized prior to sample preparation. Duck breast meat and seasonings were obtained from Charoen Pokphand Supermarket (C. P. Group, Changsha, China), with the same production batch and slaughter batch. The breed of duck was Cherry Valley duck. A total of 20 duck breast fillets were used in this study, with each breast weighing 300 ± 50 g. After thawing in a refrigerator at 4 °C, the pectoralis major muscle located at the anterior part of the sternum were separated, and the portion of pectoralis major near the pectoralis minor and keel of duck were discarded. Then skin, connective tissue, fat, and muscle membranes were excised and removed to get a smooth surface of duck meat. Then meat was cut into uniform cubes of 2*2*2 cm^3^. The pre-cooking process was conducted by braising with spices for 30 min, following the method reported by Wang et al. (14). For short, add 1% salt, 1% sugar, 0.5% chili powder, 0.5% soy sauce, 4% cooking wine, and 1.5% spices to 1,500 g of pure water. Boiling at 100 °C for 30 min, then let it stand for 15 min to get the braised soup. Place 500 g of duck meat into 1,500 g of the braised soup, stewing at 90–100 °C for 30 min, and let it stand for 15 min to complete the process. This process for precooked duck meat is a commonly applied and accepted method though Chinese ready-to-eat meat markets. Spices were provided by Juewei Food Co., Ltd.

### Quick freezing methods

**Figure 1:**
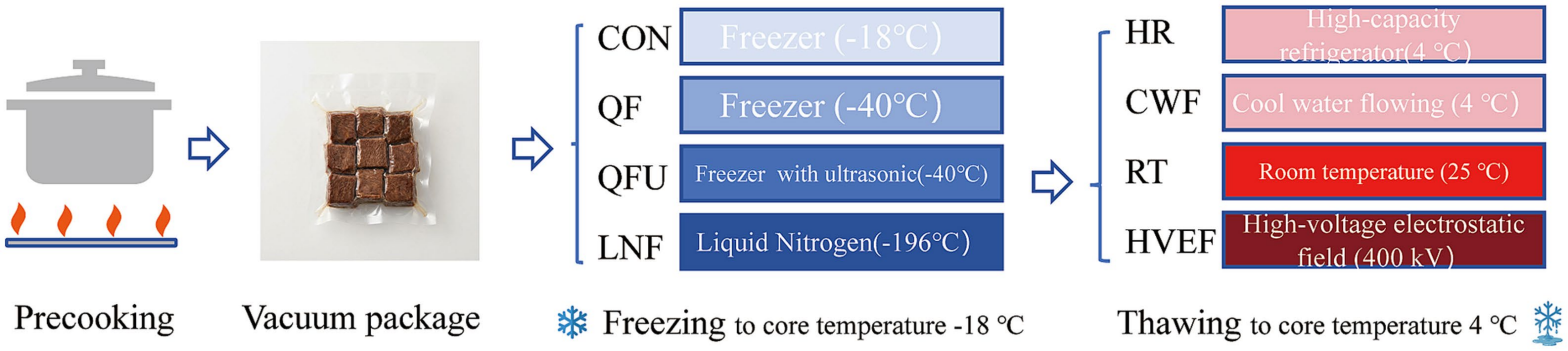
The preparation of precooked duck samples.

Following cooling to room temperature (25 ± 5 °C), the precooked duck meat samples were vacuum-packed in PP (polypropylene) bags with a vacuum degree of 0.1 ± 0.02 MPa. The PP bags are impermeable to both water and air, ensuring airtight and watertight sealing during freezing treatments. Then samples were allocated to four freezing treatment groups randomly (Figure 1), including (1) −18 °C freezing group (CON), in which meat samples were frozen by a freezer (HCF-460HTQ, Haier, China); (2) quick freezing group (QF), in which meat samples were frozen by a quick-freezing equipment (KBQ16PA, Barhe, China), powered by compressors provides −40 °C condition (N-R404, SECOP, Germany); (3) The QFU treatment was applied using an ultrasonic-assisted quick-freezing system (KBQ16PA, Barhe, China) equipped with ultrasonic transducers (200 kW, 45 Hz). The samples were immersed in a freezing solution composed of (w/w): pure water as the base, 20% ethanol, 10% propanediol, 7% betaine, and 10% sodium chloride, on a total dipping solution weight basis. The ethanol and propanediol serve as cryoprotectants to reduce the freezing point; betaine acts as an osmotic regulator and protein stabilizer; sodium chloride maintains ionic strength and enhances heat transfer; (4) liquid nitrogen freezing group (LNF), in which a liquid nitrogen spray is used to maintain the temperature of the freezing chamber at −80 °C (KLS-XSD-10, CRYOSTECH, Chengdu, China). A multi-path temperature tester (TP9000, TOPRIE, Shenzhen, China, T-type thermocouples with Teflon-coated probes, TT-T-36, RUIKELI, Jiangsu, China) was used to record changes in the core temperature and freezing time of each sample at 1 s intervals.

### Thawing process

Four thawing methods were compared, including three conventional and commercial approaches and one novel method: (1) High-capacity refrigerator (HR), where precooked meat samples with an initial core temperature of −18 °C were thawed in a freezer (HCF-460HTQ, Haier, China); (2) Cool water flowing (CWF), where precooked meat was thawed using 4 °C cool water flow; (3) Room temperature (RT), where precooked meat was thawed at 25 °C; (4) High-voltage electrostatic field (HVEF) was generated by a high-voltage electrostatic generator (model YHZF-400KV/5 mA, resolution 0.1% kV, SUHAI, Shanghai, China), where precooked meat was thawed using a high-voltage electrostatic field at 400 kV. Before undergoing the different thawing methods, all samples were taken out from vacuum packaging bags. Thawing was terminated once the core temperature of the samples reached 4 °C.

### Centrifugal loss

The centrifugal loss measurement was conducted according to Li et al. (15). The meat samples were thawed, wrapped with filter paper and weighed (M_0_ g). Then meat was centrifuged at 4000 rpm, 4 °C for 30 min. Subsequently, meat was wrapped with filter paper and reweighed (M_1_ g). The centrifugal loss was calculated with the Equation 1:

$$\text{Centrifugal loss}\;(\%)=(M_{0}-M_{1})/M_{0}*100\%$$ (1)

### Color

The color of meat was measured with a chroma meter (CR-400, Konica Menonda, Tokyo, Japan). The detection parameters were L* (brightness), a* (greenness) and b* (yellowish-blue). Before measurement, chroma meter was calibrated using a white porcelain calibration plate (Y-value of 93.60, x-value of 0.3134, and y-value of 0.3194).

### Texture profile analysis (TPA)

The TPA was examined following the conditions that described previously Kang et al. (16). For short, texture profile including hardness, adhesiveness, cohesiveness, resilience, gumminess and chewiness were measured by a TMS-Pilot Food Texture Analyzer, and calculated by TPA program inside (Food Technology Corporation, Stering, VA, United States). TPA probe model was P50, diameter = 50 mm, and parameters were set with pretest speed = 120 mm/min, test speed = 60 mm/min, posttest speed = 120 mm/min, interval time = 5 s, trigger force = 5 g and compression ratio = 50%.

### Total sulfhydryl content

Total sulfhydryl groups measurement was performed using Ellman’s method (17). Meat sample was cut into pieces, with fascia and fat removed, homogenized, myofibrillar protein extracted with PBS; centrifuged at 4 °C, 8000 g for 15 min, precipitate washed twice, dissolved in purified water, centrifuged at 13000 g for 5 min to collect the supernatant. Mix 0.5 mL of a 1 mg/mL sample supernatant solution with 2.5 mL of Tris-Gly-8 M urea buffer (pH 8.0) and 0.02 mL of a 4 mg/mL DTNB solution. Incubate the mixture at 25 °C for 30 min, then measure the absorbance at 412 nm.

### Carbonyl content

Carbonyl content was determined using the Protein Carbonyl Content Assay Kit (Sigma-Aldrich, MAK094) as follows: The spectrophotometer was preheated for 30 min, wavelength set to 375 nm. A 96 well Plate was used, and 100 μL of sample supernatant and 100 μL DNPH solution were added to each well, vortexed and incubated in the dark for 10 min; 30 μL 87% TCA Solution was added, and placed in an ice bath for 5 min, centrifuged at 4 °C, 13000 g for 2 min, supernatant discarded; washed twice with 500 μL ice-cold acetone, 200 μL 6 M guanidine solution was added for dissolution, 100 μL aliquot taken to measure A375. The 5 μL sample was used to determine protein concentration by the bicinchoninic acid (BCA) assay. Calculate the carbonyl content according to the Equation 2:

$$\text{Carbonyl content}\;(\text{nmol}/\mathrm{mg}\;\text{protein})=\frac{(A_{1}-A_{2})/6.354}{\mathrm{Cpr}}\times 1000\;$$ (2)

A1 denotes the absorbance of the assay well, A2 represents the control absorbance of well, and Cpr indicates the protein concentration of the sample.

### Thiobarbituric acid reactive substances content

The concentration of TBARS was determined using a slightly modified method of Tarladgis et al. (18). Two grams of meat sample was minced, mixed with 3 mL of 1% thiobarbituric acid (TBA) and 17 mL of 2.5% trichloroacetic acid-HCl (TCA-HCl), and supplemented with 0.5 mL of 0.19 M butylated hydroxytoluene (BHT). The mixture was heated in a boiling water bath at 100 °C for 30 min. After cooling to 25 °C, 4 mL of the resulting suspension was mixed with 4 mL of chloroform, vortexed for 1 min, and centrifuged at 3000 rpm for 20 min. The absorbance of the supernatant was measured at 532 nm.

### Scanning electron microscope and ice crystal size

Cubes of meat (1 * 5 * 5 mm) were cut perpendicular to the muscle fiber and fixed with glutaraldehyde buffer solution for 4 h. Then meat was eluted with 10, 30, 50, 70, 80, 90, and 100% alcohol for 10 min, respectively. After freeze-drying for 24 h and 4 Pa, the meat samples were sprayed with gold (1.5 kV, 30 mA, 2 min), and the microstructure of the muscle was observed using scanning electron microscopy (JSM-5410, Jeol, Tokyo, Japan) at an acceleration voltage of 20 kV.

### Low-field nuclear magnetic resonance (LF-NMR)

Meat samples were sliced parallel to the direction of intramuscular fibers (1 cm × 1 cm × 3 cm) and secured in glass NMR tubes. Prior to analysis, the samples were equilibrated thermally at 25 °C for 30 min. The Carr-Purcell-Meiboom-Gill (CPMG) pulse sequence was employed to determine the transverse relaxation time (T₂), with instrument parameters configured as follows: 22.6 MHz proton resonance frequency; 25 mm radio frequency coil diameter; 4,000 ms repetition delay; 15,000 echoes collected at 0.25 ms intervals; 16 scan repetitions. The decay curves were analyzed using MultiExpInv Analysis software (Niumag Electric Corporation, China).

### Statistical analysis

The experimental design was completely randomized, and each trial was repeated for at least three times. Measurements for each sample were also repeated at least three times. Data are presented as mean ± SD. Statistical analysis was conducted using Origin 2020 Pro software, applying one-way analysis of variance (ANOVA) and followed by Tukey’s significant difference test (*p <* 0.05).

## Results and discussion

### Freezing rate and water holding capacity

**Figure 2:**
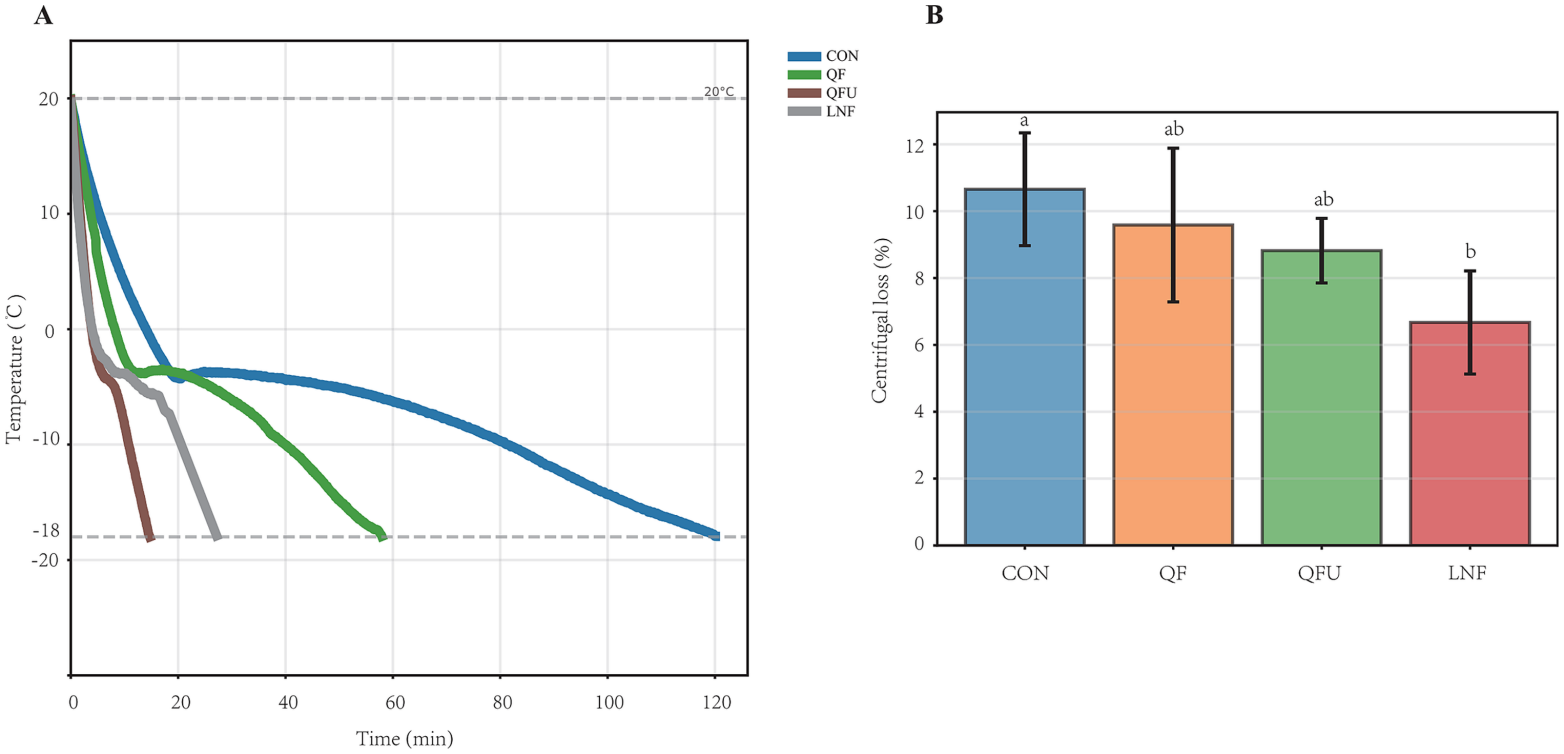
**(A)** The core temperature of meat under different freezing methods. **(B)** Centrifugal loss of meat under different freezing methods.

Figure 2 presents the effects of −40 °C combined with ultrasonic on freezing time and freezing losses. The freezing rates for CON, QF, QFU and LNF are 1, 2.07, 4.41 and 8.16 cm/h, respectively (Figure 2A). To replicate test of industrial conditions, the power of the commercial liquid nitrogen quick-freezing equipment was restricted. Meat samples of LNF were cooled by a nitrogen sprayer rather than immersing in liquid nitrogen directly. The QF treatment was operated at a refrigeration power of 4 kW. Under these conditions, the time required for the QF process to reduce the sample’s core temperature to −18 °C was shortened 7% compared with CON. When QFU of 200 W was applied, the freezing rate of the sample’s core temperature between 20 °C and −2 °C was similar to that freezing by liquid nitrogen. Studies have indicated that ultrasound enhances the heat transfer rate and facilitates ice crystal nucleation, reducing the freezing time, typically within the range of −0.5 °C to −2.8 °C (19). This process promotes the growth of crystals rather than the formation of larger crystal nuclei. Despite rapid cooling rate compared with the QF, which showed a significantly steeper cooling curve slope between −2 °C and −18 °C, the QFU group exhibited discernible temperature fluctuations. These findings indicated that ultrasound enhanced both heat and mass transfer, thereby reducing freezing time. However, the temperature fluctuations may be attributed to localized heating effects resulting from ultrasound cavitation and the accompanying thermal dynamics during the freezing process (20). Ultrasound treatment increased the freezing rate, but it also generated heat during the process, which could reduce the freezing rate if high power levels are applied. In the future, sensors can be used to reduce the thermal effects of ultrasound, ensuring that the heat generated is absorbed by the refrigerant rather than by the precooked meat.

Centrifugal loss is a key parameter for evaluating the water-holding capacity of fresh or cooked meats, as it reflects moisture expelled during storage, processing, and cooking. In present study, different freezing rates significantly influence the centrifugal loss of precooked meat, in which LNF decreased centrifugal loss by 37.4% (6.67% vs. 10.65%, *p <* 0.05), while the quick freezing with ultrasonic (QFU) reduced it by 17.2% compared to CON (8.82% vs. 10.65%, Figure 2B). The centrifugal loss in cooked meat products is contingent upon the retention of fat and water, with the mobility of these constituents following *Darcy*’s law and influenced by contraction pressure (21). These factors have a direct effect on the flavor and texture of precooked meat. Consequently, the rapid freezing with ultrasonic partially decreased the mobility of fat and moisture, thereby enhancing the quality characteristics of precooked meat.

### Color analysis

The acceptability of meat products to consumers is significantly influenced by their color and appearance. The pH, L*, a*, and b* values are presented in Table 1. Compared with QF, LNF significantly reduced the pH value of meat (*p <* 0.05). Generally, a decrease in pH following freezing and thawing of meat may be attributed to the denaturation of buffering proteins, the release of hydrogen ions, or the loss of fluids containing concentrated solutes (21). In this study, differences in freezing rates had no significant effect on the L*, a, or b color values of precooked meat (*p* > 0.05). The color of different precooked meats is influenced by fat and protein content. It has been reported that the correlation between color and temperature in lean differed from fatty meat is inconsistent across freezing temperatures, indicating that not all freezing rates effectively preserve color (22). Xu et al. (23) reported that L* showed no significant variation between ultrasonic pretreatments in vacuum-cooled chicken breast, which was similar with present study.

**Table 1:** Effects of freezing methods on the color and pH of precooked meat.

Freezing systems^1^^,^^2^	CON	QF	QFU	LNF
L	44.15 ± 1.26	44.54 ± 1.41	43.66 ± 1.76	43.98 ± 1.21
a	11.26 ± 1.17	11.12 ± 1.15	11.65 ± 0.7	11.02 ± 1.62
b	17.23 ± 1.2	17.1 ± 1.42	17.23 ± 1.32	17.03 ± 2.58
δE		2.36 ± 0.61	2.01 ± 0.92	2.7 ± 1.11
pH	6.047 ± 0.012^ab^	6.053 ± 0.006^a^	6.023 ± 0.006^b^	6.03 ± 0.01^b^

^1^CON, slow freezing group; QF, quick freezing group; QFU, quick freezing with ultrasound; LNF, liquid nitrogen freezing group. ^2^The data were expressed as the mean ± standard deviation. ^a, b^Means with different superscripts within same column are significantly different (*p <* 0.05).

### Texture profile

The texture of meat products is closely related to their microstructural organization and physical properties, both of which influence the eating quality. Table 2 shows the effects of freezing methods on TPA of precooked meat. Compared to CON, the QF, QFU, and LNF with faster freezing rates reduced the shear force, resilience, and chewiness of precooked meat. Among these, QF significantly decreased the shear force (*p <* 0.05), while ultrasound treatment partially restored the shear strength. Previous studies have shown that ultrasound-assisted freezing of stewed beef at 400 W can significantly improve tenderness, reducing hardness from 5601.50 g to 2849.46 g (24). Additionally, other studies have demonstrated that improving freezing rate significantly influenced the TPA parameters of pork leg muscle (25), which was similar with the present results. When ultrasound is assisted in thawing of meat, the improved textural characteristics are generally attributed to the acoustic cavitation generated at ultrasonic frequencies (26). This process may promote liquid in the part of myofibrillar and intermyofibrillar space to form additional cavitation nuclei, when ultrasound is applied to freezing, which affects TPA of precooked meat eventually. However, the application of ultrasound-assisted freezing remains limited, with few studies specifically addressing meat freezing, despite the potential of ultrasound to reduce ice crystal size in food.

**Table 2:** Effects of freezing methods on TPA of precooked meat.

Freezing systems^1^^,^^2^	CON	QF	QFU	LNF
Shear force/N	11.18 ± 1.92^a^	9.12 ± 2.24^b^	9.99 ± 1.86^ab^	9.34 ± 1.70^b^
Hardness/g	2610.6 ± 627.7	2249.8 ± 678.6	2138.8 ± 896.0	2476.9 ± 419.9
Cohesiveness/g	0.38 ± 0.06	0.37 ± 0.02	0.38 ± 0.07	0.37 ± 0.05
Resilience/mm	6.49 ± 0.40 ^a^	5.98 ± 0.29 ^ab^	5.60 ± 0.67 ^b^	5.44 ± 0.48 ^b^
Gumminess/g	960.5 ± 154.4	816.9 ± 227.6	785.7 ± 242.9	917.0 ± 104.7
Chewiness/mJ	61.29 ± 11.67 ^a^	48.00 ± 14.24 ^ab^	42.23 ± 11.24 ^b^	48.66 ± 4.67 ^ab^

^1^CON, slow freezing group; QF, quick freezing group; QFU, quick freezing with ultrasound; LNF, liquid nitrogen freezing group. ^2^The data were expressed as the mean ± standard deviation. ^a, b^Means with different superscripts within same column are significantly different (*p <* 0.05).

### Protein and lipid oxidation

**Figure 3:**
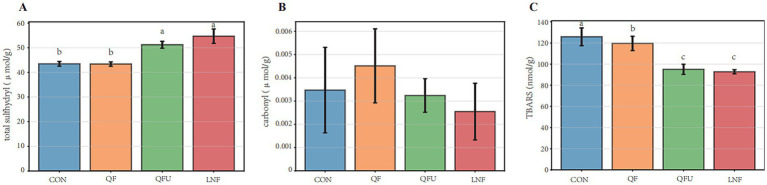
Protein and lipid oxidation of meat under different freezing methods. **(A)** Total sulfhydryl. **(B)** Protein carbonyl. **(C)** TBARS value.

The sulfhydryl content and TBARS value in meat are important indicators of freshness and eating quality. The total sulfhydryl content and TBARS value under different freezing methods are shown in Figure 3. During processing, sulfhydryl groups in meat are oxidized to disulfide bonds as temperature rises and actin conformation changes (27). In this study, the total sulfhydryl content remained higher than 40 μmol/g (Figure 3A), and QFU and LNF significantly increased total sulfhydryl content (*p <* 0.05), indicating that ultrasound preserved 17.8% unoxidized sulfhydryl. Reactive oxygen species target sulfhydryl and cause substantial losses (28), which is particularly relevant for precooked meat that requires reheating. Moreover, different freezing methods did not significantly affect protein carbonyl levels, which were low in precooked meat (Figure 3B). Furthermore, TBARS in precooked meat were also investigated (Figure 3C). The results showed that accelerating the freezing rate significantly suppressed lipid peroxidation, with QFU and LNF exhibiting the lowest TBARS value (*p <* 0.05). In the food industry, all processing methods can markedly increase MDA compared with fresh, unprocessed foods, including freezing, as well as boiling, frying, and roasting (29). Repeated freezing and thawing cycles progressively increase peroxide value, thiobarbituric acid reactive substances, non-heme iron content, and carbonyls, while decreasing sulfhydryl group content (30). Therefore, reducing protein and lipid oxidation during freezing can improve meat freshness. Kim et al. (31) reported that faster freezing rate inhibits protein and lipid peroxidation in pork, which is consistent with the present findings. Furthermore, structural characterization was conducted to evaluate the distribution of crystal nuclei in meat.

### SEM and ice crystal size

**Figure 4:**
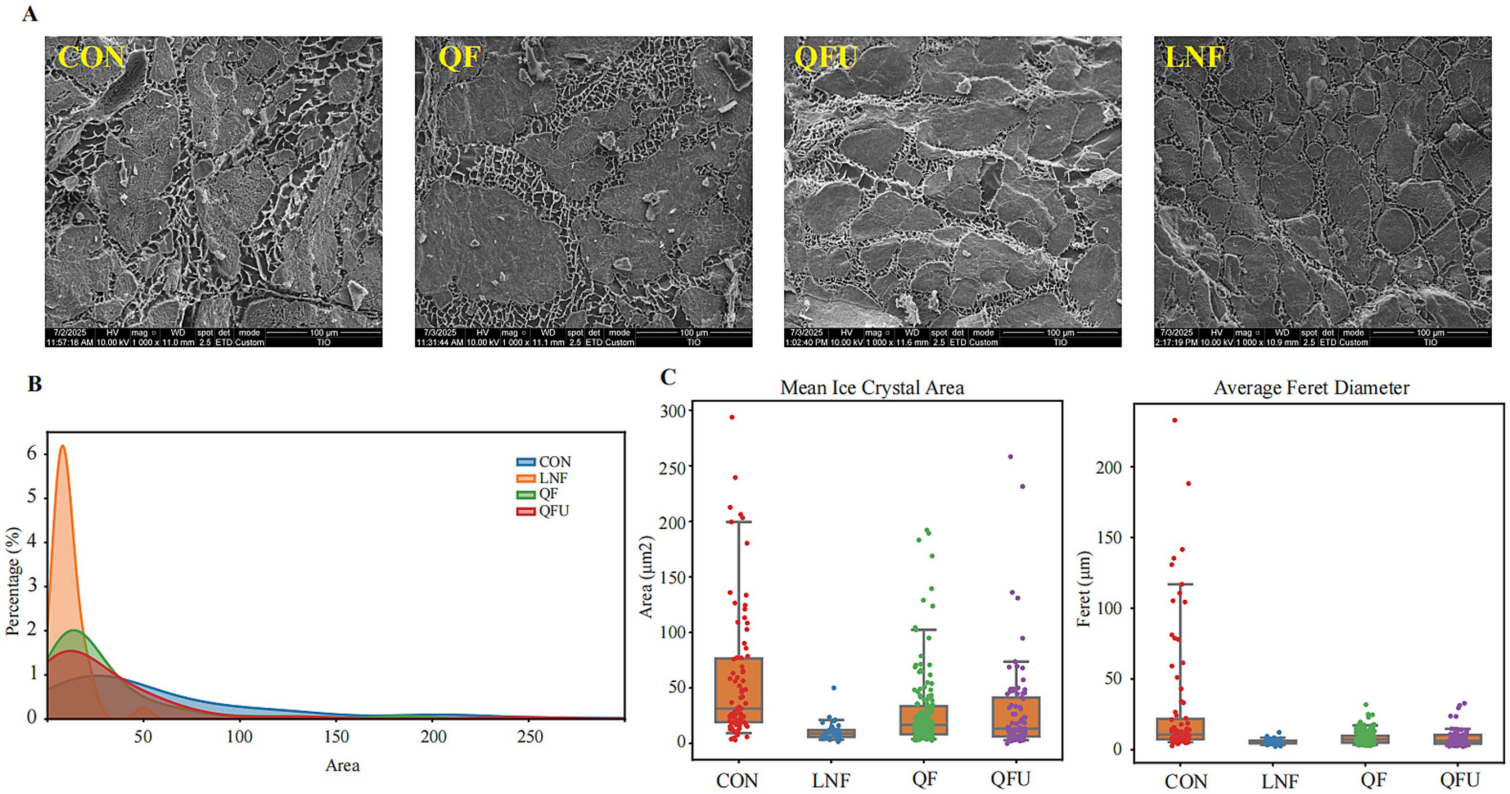
Scanning electron microscopy results of meat under different freezing methods. **(A)** Images of meat under different freezing methods (1000×). **(B)** Area fraction distribution of ice crystals. **(C)** Ice crystal area and diameter.

Scanning electron microscopy was used to investigate the microstructural changes in muscle fibers and the distribution of ice crystals under different freezing methods (Figure 4A). At a magnification of 1,000×, ice crystal sizes varied significantly across treatments. In the CON group, the formation and growth of ice crystals widened the inter-fiber spaces and caused muscle fiber separation. In contrast, the QFU and LNF groups exhibited smaller gaps and better-preserved fiber bundles, highlighting the superior protective effects of ultrasound and liquid nitrogen freezing with higher freezing rates. The area fraction of ice crystals produced by each freezing method was further quantified (Figure 4B and Supplementary Table S2). The LNF group exhibited the highest percentage of small ice crystal area overall, with the highest percentage located in 12 μm^2^, while the QF and QFU groups showed varying increases in the proportion of small ice crystals of all ice crystal formation compared with CON. Likewise, Mulot et al. (32) quantified the relationship between cooling rate and crystal size, stating that increasing the integral cooling rate from 0.9 °C/min to 4.5 °C/min decreased the mean equivalent crystal diameter from 202 to 73 μm. The heat-transfer efficiency of different meat products varies and follows Fourier’s equation. Jia et al. (33) reviewed the inhibitory effects of applying ultrasound of different intensities during freezing on ice-crystal formation in various meats. For porcine longissimus dorsi treated with 30 kHz, 180 W ultrasound, the phase transition time was the shortest, with a mean ice-crystal radius of 5.02 μm; for common carp treated with 30 kHz, 175 W ultrasound, the mean ice-crystal radius was 21.47 μm. The mean ice crystal area and the mean equivalent diameter (Feret diameter) were significantly different among the groups (Figure 4C), with the CON group displaying the largest values (*p <* 0.05). Larger ice crystals result in larger voids, which, during thawing, may facilitate the formation of water channels, thus negatively impacting the water-holding capacity of meat products (34, 35). The size of the formed ice crystals affects muscle fiber deformation and the size of inter-fiber gaps, which may explain the preservation of structure and texture in pre-cooked meat while also reducing the oxidation of flavor compounds. In addition, indirect observation techniques such as X-ray computed tomography, nuclear magnetic resonance, and visible-near-infrared spectroscopy, by exploiting the birefringent dispersion characteristics under polarized-light interference, can clearly document the growth process and morphological features of ice crystals (36, 37). This enables in-depth investigation of how ice-crystal morphology affects meat quality.

### Lf-NMR

**Figure 5:**
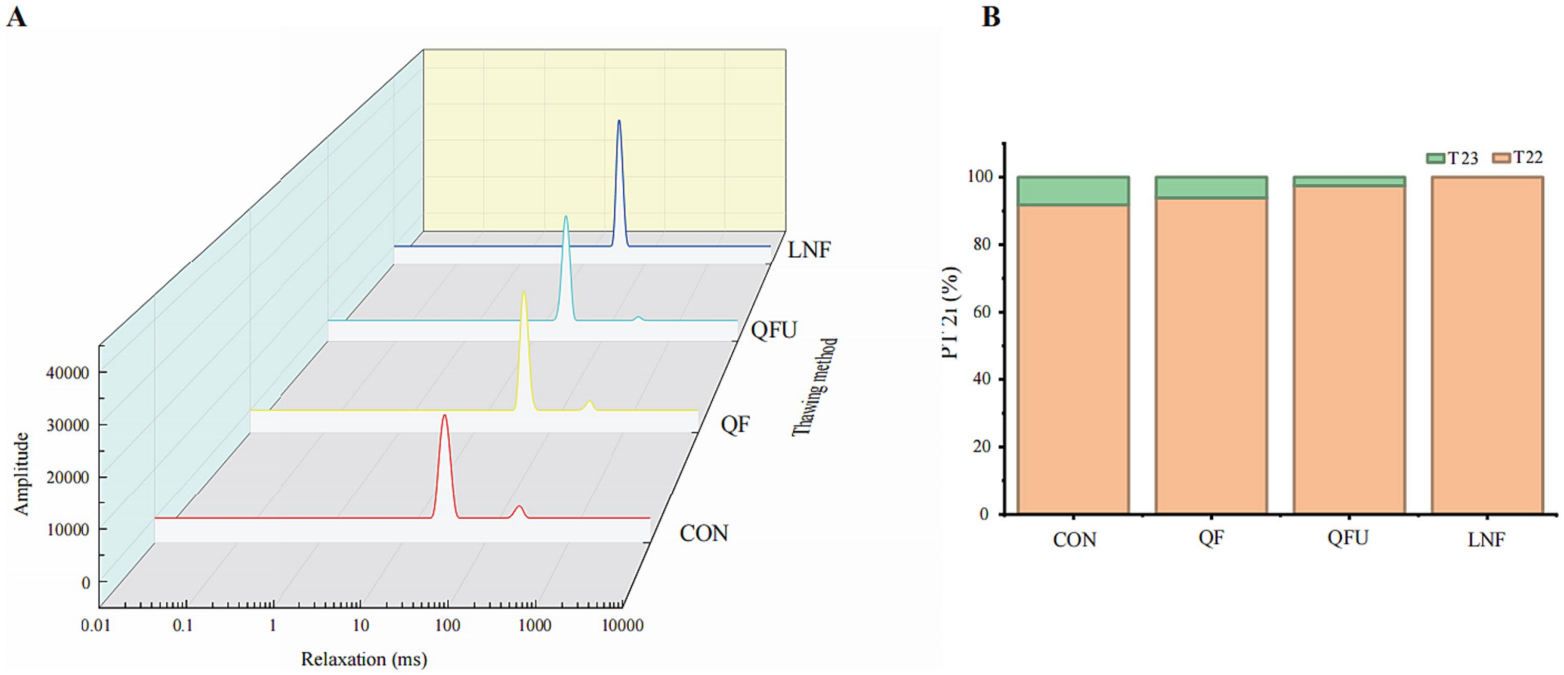
LF-NMR results of freezing methods. **(A)** Water relaxation intensity. The transverse relaxation times T_21_, T_22_, and T_23_ represent three distinct water populations in the meat matrix: T_21_ (10–100 ms) corresponds to water tightly bound to macromolecules such as proteins and lipids; T_22_ (100–1,000 ms) corresponds to immobilized water within the myofibrillar protein network and cell membranes; T_23_ (>1,000 ms) corresponds to free water in the extracellular space. **(B)** Proportion of water relaxation intensity. The peak areas of T_21_, T_22_, and T_23_ were integrated to calculate the relative proportion of each water population.

LF-NMR is a technique used to measure the physical and chemical mobility of water in samples and to investigate properties of the meat matrix, such as water distribution, compartmental organization, water mobility (38). In this study, precooked meat samples with different freezing methods exhibited two peaks, in which the T_2b_ relaxation intensity was absent (Figure 5A). The water populations were present in T_21_ and T_22_, indicating that in the precooked meat, there were no strongly bound water molecules. When precooking, the helical spatial structures of proteins are disrupted, and hydrophilic groups, such as hydroxyl and amino groups, lose their capacity to bind water. As a result, water evaporates as steam or is lost with meat juices during heating (39, 40). The 10–100 ms T_21_ relaxation intensity contributed more than 90% of the total signal intensity (Figure 5B), most likely reflecting water located within highly organized protein structures, such as tertiary and quaternary structures associated with high myofibrillar protein density, and the actin and myosin filament structures. This range is also recognized as the fast relaxation component. In addition, T_22_ had varying signal intensities. As the freezing rate increased, the proportion of the T_22_ in precooked meat decreased. Compared with CON and QF, the QFU samples freezing with ultrasound exhibited the lowest T_22_ proportion. Interestingly, T_22_ in LNF was undetectable, indicating that rapid freezing with liquid nitrogen reduced the free water content in the meat, which is consistent with a previous study that reported that LNF at −95 °C decreases water mobility (41). In summary, QFU reduced the T_22_ fraction and modulated the moisture distribution in precooked meat, with a rapid freezing rate.

### Thawing methods on quality of precooked meat

**Figure 6:**
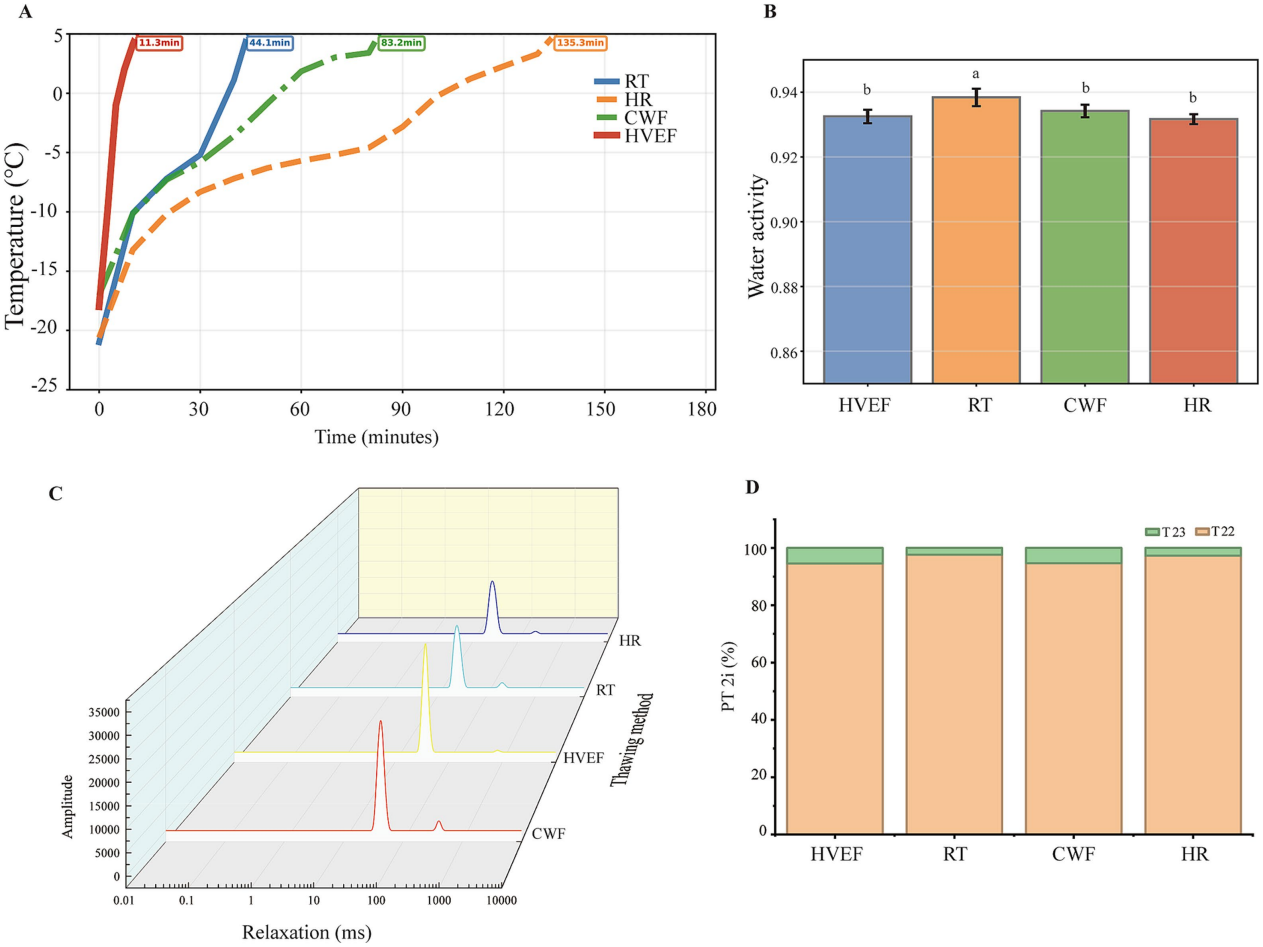
Effects of thawing method on quality of precooked meat. **(A)** Core temperature cooling rate during thawing. **(B)** Water activity (Aw) of precooked meat after thawing. **(C)** LF-NMR water relaxation intensity (T₂ distribution) after thawing. **(D)** Proportion of water relaxation intensity. Different lowercase letters indicate significant differences (*p <* 0.05). T_21_: bound water; T_22_: immobilized water; T_23_: free water.

The impact of combining the novel technology HVEF thawing with QFU freezing on the quality of precooked meat was investigated (Figure 6). RT, CWF, and HR are common thawing methods with mild heat-exchange rates, which were commonly used to thaw precooked meat. With HVEF, the cooling rate of precooked meat samples was the fastest (Figure 6A). The thawing rates for HVEF, RT, CWF, and HR were 10.62, 2.72, 1.44 and 0.89 cm/h, respectively. Differs from the conventional heat-transfer thawing where energy is transferred through conduction and convection at the surface, HVEF thawing operates through two primary mechanisms. Ionic wind generation occurs when high voltage is applied between electrodes, ionizing and accelerating air molecules, which creates a continuous momentum transfer that enhances energy exchange at the meat surface (42). The polarization of water molecules is enhanced by the electrostatic field, which lowers the energy barrier for the ice-to-liquid phase transition and reduces the duration of the critical temperature zone. This explains why HVEF thawing operates effectively at low ambient temperatures without inducing dielectric heating. In contrast to microwave thawing, this characteristic of HVEF thawing prevents protein denaturation. Different thawing methods had no effect on centrifugal loss (*p* > 0.05, Supplementary Figure S1). Thawing methods significantly affected meat color (Supplementary Table S1). The pH values of HR and CWF samples were higher than those thawed by RT (*p <* 0.05). The L, a, and b values of HVEF thawing and RT samples were significantly lower than those of HR (*p <* 0.05). Hence, HVEF and RT thawing precooked meat resulted in a darker meat color.

Table 3 showed the effects of thawing methods on the TPA of precooked meat. After HR thawing, the shear force of precooked meat was significantly lower than that of the other three thawing methods (*p <* 0.05). The hardness of precooked meat was the lowest after RT thawing (*p <* 0.05). The four thawing methods have no significant effects on cohesiveness, resilience, gumminess, or chewiness of precooked meat. The effect of thawing method on texture can be understood through the lens of ice-water coexistence duration. During thawing, as the temperature passes through the −5 °C to 0 °C range, ice crystals melt while the protein matrix remains partially frozen. Additionally, slow thawing exacerbates protein network relaxation and drip loss, resulting in softer texture (43). In contrast, HVEF thawing minimizes the duration of ice-water coexistence, thereby limiting structural disruption to the cooked protein matrix. The accelerated thawing rate of HVEF may have preserved the integrity of the protein gel network formed during cooking (44). The lower shear force in HR-thawed samples may therefore reflect greater protein network disruption rather than a desirable tenderization effect. Previous studies have mainly focused on the effects of freezing and thawing methods on the texture of raw meat. For example, Ramos et al. (45) reported that steaks frozen in a commercial freezer and subsequently thawed for 24 h exhibited decreased tenderness due to cold injury-induced cellular damage rather than an increased proteolysis rate. In present study, muscle fibers underwent irreversible contraction after cooking, and voids left by ice crystals in damaged fibers would also irreversibly enlarge due to heat-induced shrinkage (46). Therefore, the heat transfer effects of different thawing methods on water influenced the textural properties of precooked meat. In addition, salt may affect structural stability during the freezing and thawing process of precooked meat (47). According to Raoult’s law, interactions formed between solvent and solute allow sodium and chloride ions to be attracted to the negative and positive poles of water molecules within muscle fibers, thereby weakening intermolecular interactions among water molecules, preventing the formation of stable hydrogen bonds (48). This ionic solvation effect disrupts the organized water structure around protein molecules, increasing the mobility of unbound water and destabilizing the protein-water interactions that are essential for maintaining the gel network integrity formed during cooking. When thawing, the depressed nucleation temperature caused by these salt-water interactions prolongs the duration of the ice-liquid coexistence zone, during which the weakened protein-water interactions are particularly susceptible to disruption by migrating ice crystals. A slower thawing rate extends this vulnerable period, allowing the salt-disrupted water structure to reorganize in a manner that favors water migration out of the tissue and further loosens the protein matrix. Therefore, a faster thawing rate in this study, particularly under HVEF thawing, reduced the time available for this structural destabilization to occur, thereby preserving the textural properties of precooked meat.

**Table 3:** Effects of thawing methods on TPA of precooked meat.

Thawing systems^1^^,^^2^	HVEF	RT	CWF	HR
Shear force/N	13.49 ± 4.78^a^	12.55 ± 2.66^a^	13.17 ± 2.77^a^	8.93 ± 1.84^b^
Hardness/g	2263.5 ± 410.4^a^	1602.8 ± 388.6^b^	2199.8 ± 534.8^a^	2106.8 ± 565.1^a^
Cohesiveness	0.41 ± 0.07	0.43 ± 0.05	0.42 ± 0.05	0.41 ± 0.06
Resilience/mm	6.24 ± 0.29	6.24 ± 0.51	6.25 ± 0.26	6.20 ± 0.49
Gumminess/g	1134.1 ± 171.7	864.0 ± 190.9	1142.2 ± 271.3	1081.66 ± 275.9
Chewiness/mJ	69.38 ± 10.47	52.70 ± 12.33	70.14 ± 17.60	65.38 ± 16.68

^1^HVEF, high-voltage electrostatic field; RT, room temp at 25 °C; cool water flowing at 4 °C; household refrigerator at 4 °C. ^2^The data were expressed as the mean ± standard deviation. ^a, b^Means with different superscripts within same column are significantly different (*p <* 0.05).

The water activity and distribution of precooked meat with different thawing methods were evaluated. Compared with RT, both HVEF and HR thawing significantly reduced the water activity of precooked meat (*p <* 0.05; Figure 6B). The application of a high-voltage electric field may have promoted the release of exudate from precooked meat, thereby altering water migration behavior and subsequent reabsorption (49).

This could be explained by the concept of Ostwald ripening: during slow thawing, small ice crystals melt and refreeze onto larger crystals, enlarging water channels and promoting water migration out of the tissue (50). Faster thawing methods reduce the time window for this process, thereby limiting water redistribution and maintaining a more compact structure. Moreover, the reduction in water activity may have contributed to the increased hardness of precooked meat (51). Figure 6C illustrated the moisture distribution of precooked meat subjected to different thawing methods. In precooked meat thawed by HVEF, the T_23_ water relaxation time was closer to 1,000 ms but represented a smaller proportion of the total water fraction (Figure 6D). The LF-NMR results further indicate that the lower T23 fraction in HVEF thawing samples suggests a reduced presence of free water, pointing to stronger protein-water interactions and improved preservation of immobilized water. Compared with HR, the T_23_ fraction increased in precooked meat thawed by RT and CWF, indicating a greater proportion of water existing as free water. In conclusion, utilizing HVEF to thaw precooked meat is a promising alternative strategy for HR, which achieves a thawing rate more than tenfold higher while maintaining superior water-holding capacity. The accelerated thawing not only improves industrial efficiency but also preserves product quality by reducing ice crystal-induced structural damage and maintaining the immobilized water fraction that is critical for textural quality in precooked meat.

## Conclusion

This study evaluated the effects of individual freezing and thawing methods, as well as their synergistic combination, on the quality attributes of precooked meat. Results showed that quick freezing with ultrasonic reduced centrifugal loss by 17.2% compared with −18 °C freezing. Freezing methods had no significant effect on the L*, a, or b color values of precooked meat. Freezing methods with faster freezing rates reduced the shear force, resilience, chewiness and TBARS of precooked meat, while improving total sulfhydryl content. SEM showed the ice crystal formation in precooked meat samples, with liquid nitrogen freezing having the highest percentage of ice crystal area <12 μm^2^. Precooked meat freezing by liquid nitrogen only showed signal intensity of T_21_, and quick freezing with ultrasonic reduced the T_22_ fraction and modulated the moisture distribution in precooked meat, with a rapid freezing rate. However, meat in the temperature range between −2 and −18 °C, the QFU group exhibited discernible temperature fluctuations, which indicated that heat generating of ultrasonic needs further research. Furthermore, four thawing methods with different rates were investigated. The HVEF thawing reduced the meat color and hardness, while having no effects of other TPA values. Compared with thawing with refrigerator, HVEF increased content of T_23_. This study confirms the critical role of the freezing and thawing methods in quality during the processing of meat prepared dishes. In summary, the combined QFU + HVEF process offers a synergistic quality-preservation strategy for the precooked meat cold chain. Rapid freezing (QFU at 4.41 cm/h) minimizes ice crystal damage and oxidative deterioration, while rapid thawing (HVEF at 10.62 cm/h) reduces the duration of the critical ice-water coexistence zone, together suppressing quality loss at both the freezing and thawing stages. Meanwhile, it provides valuable insights into the optimization of freezing and thawing processes for precooked meat dishes and offers a theoretical basis for the industrial level production of precooked meat dishes.

## Data Availability

The original contributions presented in the study are included in the article/Supplementary material, further inquiries can be directed to the corresponding authors.
