# Supplementary figures and images for: Effects of ultrasound-assisted freezing and high-voltage electric field thawing on quality of precooked duck meat
Source: Front Nutr. 2026 Apr 24;13:1791662. doi: 10.3389/fnut.2026.1791662 (PMC13152759; doi:10.3389/fnut.2026.1791662)

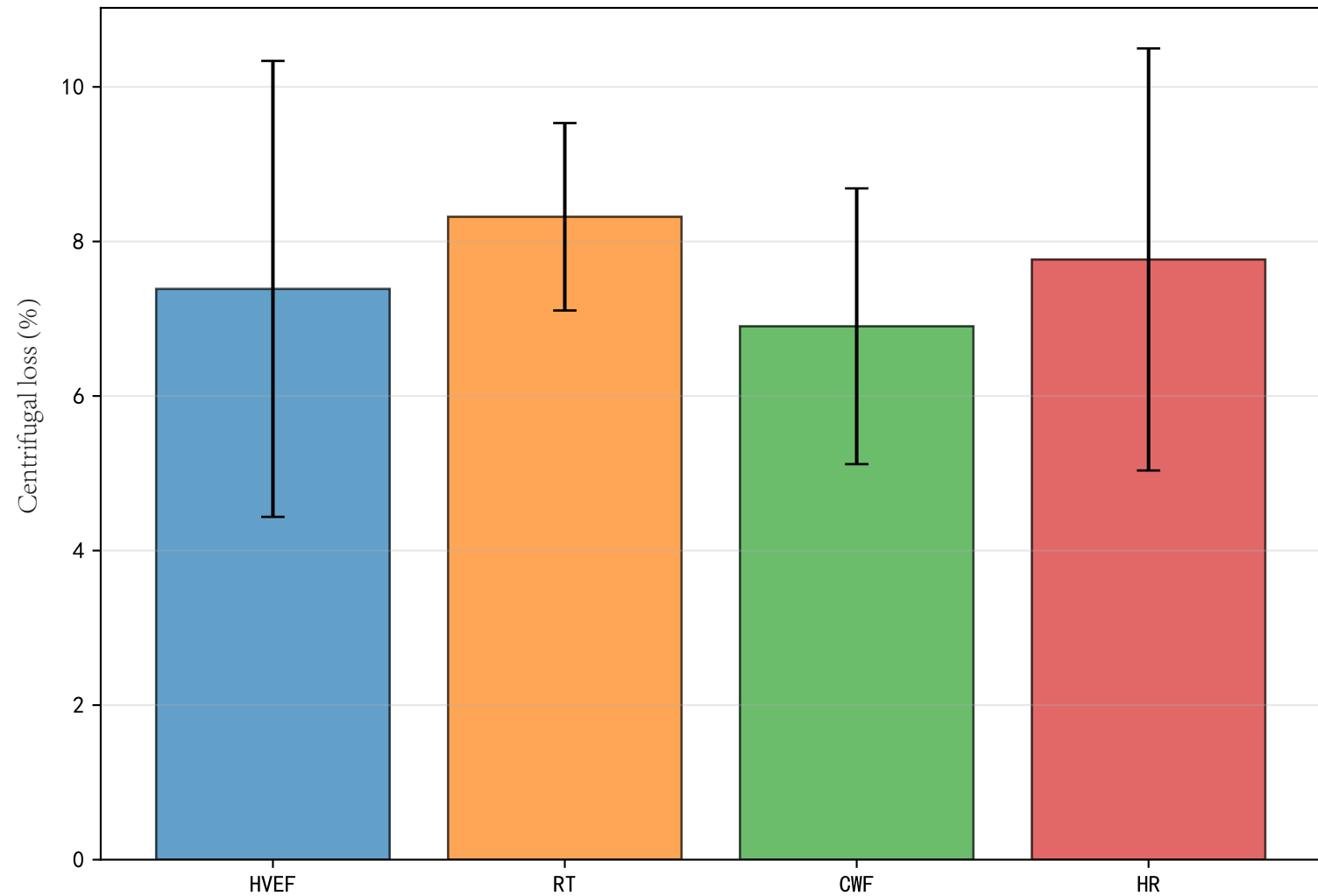

Supplement: Supplementary file 1 [file Image_1.pdf]
