# Supplementary material for: Effects of ultrasound-assisted freezing and high-voltage electric field thawing on quality of precooked duck meat
Source: Front Nutr. 2026 Apr 24;13:1791662. doi: 10.3389/fnut.2026.1791662 (PMC13152759; doi:10.3389/fnut.2026.1791662)
Supplement: Supplementary file 2 [file Table_1.docx]

Table S1 Effects of thawing methods on the color and pH of precooked meat

| Thawing systems^1)^ | **HR** | **HVEF** | **RT** | **CWF** |
| --- | --- | --- | --- | --- |
| pH | 6.070±0.010 a | 6.057±0.006 ab | 6.023±0.021 b | 6.103±0.021 a |
| L | 43.05±1.72 a | 40.83±2.48 b | 41.10±1.65 b | 42.40±1.78 a |
| a | 10.88±1.72 a | 9.65±1.38 b | 9.30±1.21 b | 10.26±1.66 b |
| b | 16.41±1.63 a | 14.98±1.48 b | 14.80±1.35 b | 16.34±1.57 a |
| δE | - | 4.96±2.45 | 4.11±2.39 | 3.90±2.03 |

1. CON, slow freezing group; QF, quick freezing group; QFU, quick freezing with ultrasound; LNF, liquid nitrogen freezing group.
2. The data were expressed as the mean ± standard deviation.

^a-b^Means with different superscripts within same column are significantly different (P < 0.05)
